# An Atypical Case of COVID-19 Induced Pancytopenia, Rhabdomyolysis and Myocarditis

**DOI:** 10.7759/cureus.12455

**Published:** 2021-01-03

**Authors:** Olusayo Fadiran

**Affiliations:** 1 Internal Medicine, Apogee Medical Group, Oregon, USA

**Keywords:** covid-19, “pancytopenia”, rhabdomyolysis, covid and myocarditis

## Abstract

Severe acute respiratory distress coronavirus 2 (SARS-CoV-2) virus is responsible for the current pandemic - coronavirus disease 2019 (COVID-19) plaguing the world. It began spreading as early as January 2020 in the United States (US) and has recently become the leading cause of death amongst adults over 45 years of age. Much of its clinical presentation is already known, and there have been advances in its successful treatment with a food and drug administration (FDA) approved antiviral medication called remdesivir, and other proven investigational methods with clinical benefits including dexamethasone and COVID-19 antibody transfusion called convalescent plasma therapy. However, the recommendations for their use include COVID-19 confirmed patients requiring supplemental oxygen or other forms of respiratory support. In this case report, we describe in detail a unique case of severe COVID-19 infection that did not require any form of oxygen support but was treated successfully with antiviral medications and steroids. The purpose of this report is to highlight in detail an unusual COVID-19 presentation with rhabdomyolysis, myocarditis, and pancytopenia severe enough to require hospitalization and treatment with proven COVID-19 therapy to achieve clinical resolution.

## Introduction

A cluster of pneumonia cases occurred in Wuhan, China, in December 2019 [1]. This was later identified to be a β-coronavirus and renamed to severe acute respiratory syndrome coronavirus 2 (SARS-CoV-2) by the Coronavirus Study Group of the International Committee and the disease it causes - coronavirus disease 2019 (COVID-19) by the World Health Organization (WHO) both in February 2020. Since its inception, it has spread to involve the rest of the world particularly, the United States. There has been emerging literature on its transmission, clinical presentation, management, complications, and epidemiology. While it has evolved into an uncontrollable pandemic with devastating economic impact and relatively high mortality, some progress has been made in developing effective treatment tools for mitigating this effect.

As such, the literature for COVID-19 is constantly evolving just as knowledge of the disease process grows, and further evidence from research develops. As of December 2020, there are over 16 million cases, with over 290 thousand deaths in the US [2]. Remdesivir, a medication that interferes with viral RNA polymerase replication [3], was approved by the FDA in October 2020 and has been demonstrated in clinical trials to improve recovery time, particularly in hospitalized patients requiring oxygen supplementation [4]. Dexamethasone decreased the risk of death in critically ill hospitalized COVID-19 patients [5]. Other treatment modalities being used include plasma containing antibodies from recovered patients of COVID-19 [6] termed convalescent plasma and synthesized monoclonal antibodies called bamlanivimab, which are being developed for emergency use authorization (EUA) medication in a non-hospitalized patient with mild to moderate symptoms [7]. A COVID-19 vaccine has been approved for EUA by the FDA at the time of our report. While most of the COVID-19 symptoms have been well characterized and guide our use of medications, atypical presentations infrequently occur, and these pose a challenge as to when and how these disease phenotypes should be treated.

In our case report, we describe a unique case of COVID-19 infection with a completely unusual presentation. The patient was a 78-year-old woman who had attended a party during which she was exposed to her son, who had recently been treated for COVID-19 and was found down on the ground in her apartment for 48 hours due to profound weakness. She did not have severe pulmonary infiltrates that required oxygen support and responded to intravenous remdesivir and dexamethasone therapy to help improve profound pancytopenia. The purpose of this case report is to raise awareness of the unusual symptomatology of COVID-19 and their response to proven therapy. 

## Case presentation

The patient is a 78-year-old female with a past medical history significant for depression, hypothyroidism, bipolar disorder, dyslipidemia, and mild cognitive impairment who developed profound weakness that was so severe that she fell to the ground and could not ambulate for 48 hours until her son found her and called 911. There was no loss of consciousness or trauma to her head.

**Figure 1:**
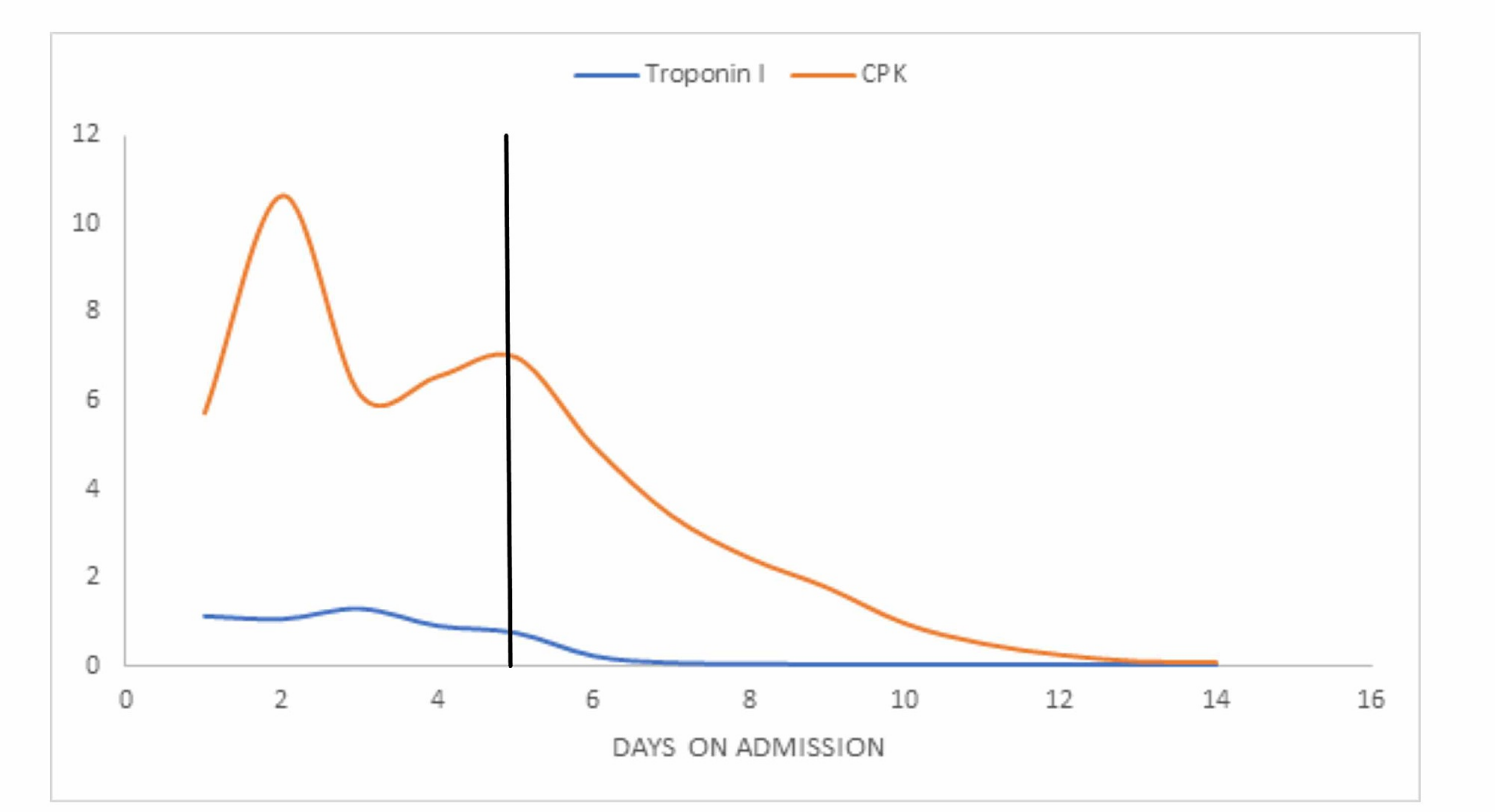
Trend in markers of skeletal and cardiac muscle injury CPK - creatinine phosphokinase, y-value x 1000units/L; Troponin I y-value in ng/mL The bold vertical line represents the onset of treatment with intravenous remdesivir and dexamethasone therapy. The x-axis represents days on admission.

**Figure 2:**
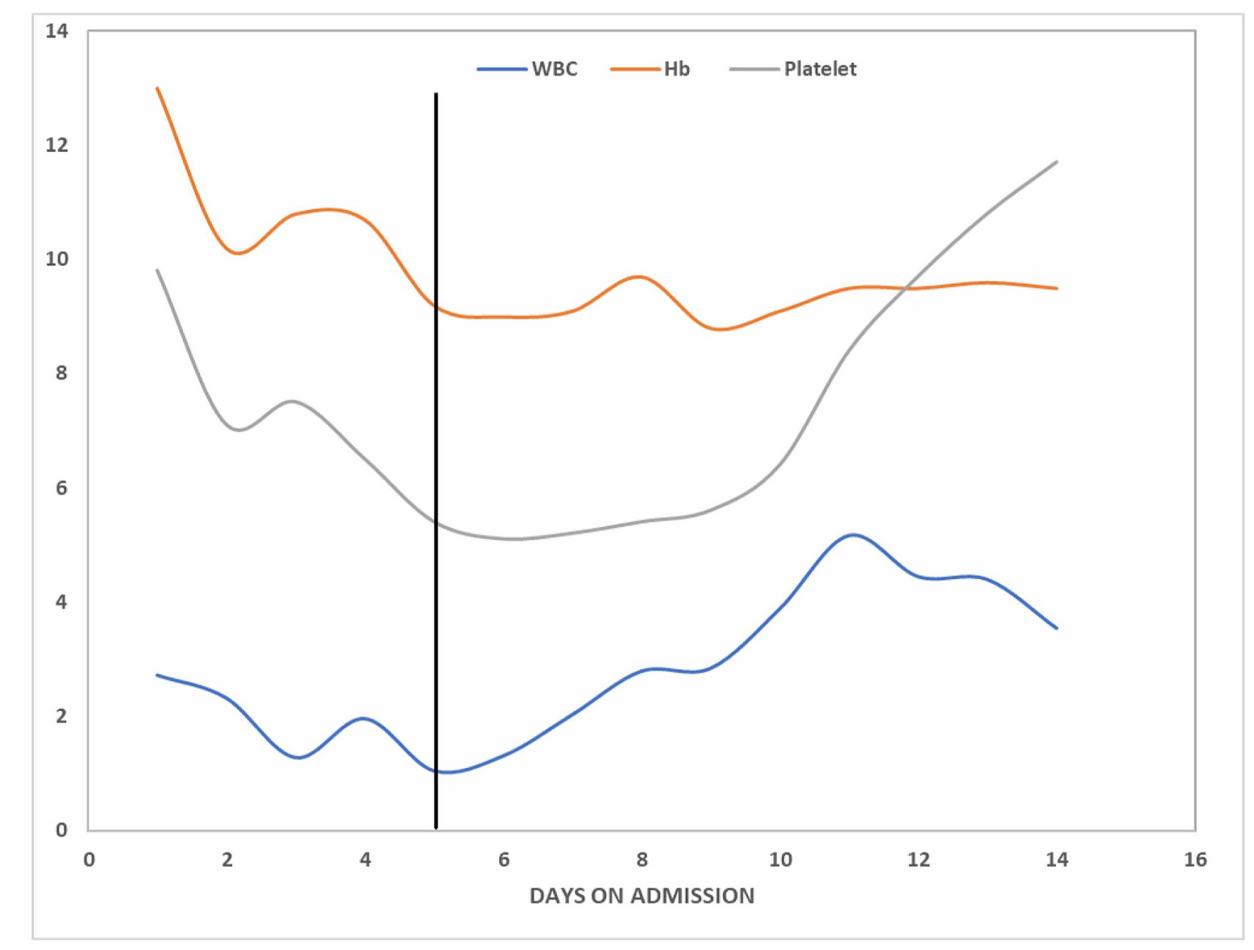
Daily trend in complete blood count WBC - white blood cell, y-value x 10^9^/L; Hb - hemoglobin, y-value in g/dL; Platelet - platelet count, y-value x 10^4^/µL The bold vertical line represents the onset of treatment with intravenous remdesivir and dexamethasone therapy. The x-axis represents days on admission.

Physical examination was unremarkable except for hypotension of 92/52mmhg, pulse rate of 110, respiratory rate of 21 breath cycles per minute, and oxygen saturation of 97% on room air. Significant laboratory workup included a positive SARS-CoV-2 test by reverse transcriptase-polymerase chain reaction via a nasopharyngeal swab, maximum creatinine phosphokinase (CPK) level of 10650units/L, myoglobin level of 1761 ng/ml, cardiac troponin I of 1.14ng/ml, elevated d-dimer of 3878ng/ml, and lactate dehydrogenase (LDH) of 550 Units/L. Complete blood count (CBC) upon presentation included a leukopenia of 2.72 X 10^9^/L, hemoglobin of 13.0g/dl, hematocrit of 37.5%, and platelet count of 98000/µL. Of note, she had a relatively normal CBC with a platelet count of 145000/µL from an outpatient workup two weeks ago. Figure 1 highlights the trend in markers of cardiac and skeletal muscle injury from admission till recovery while Figure 2 shows the daily trend in CBC elements from admission, up till the point of initiation of antiviral therapy and until recovery.

**Figure 3:**
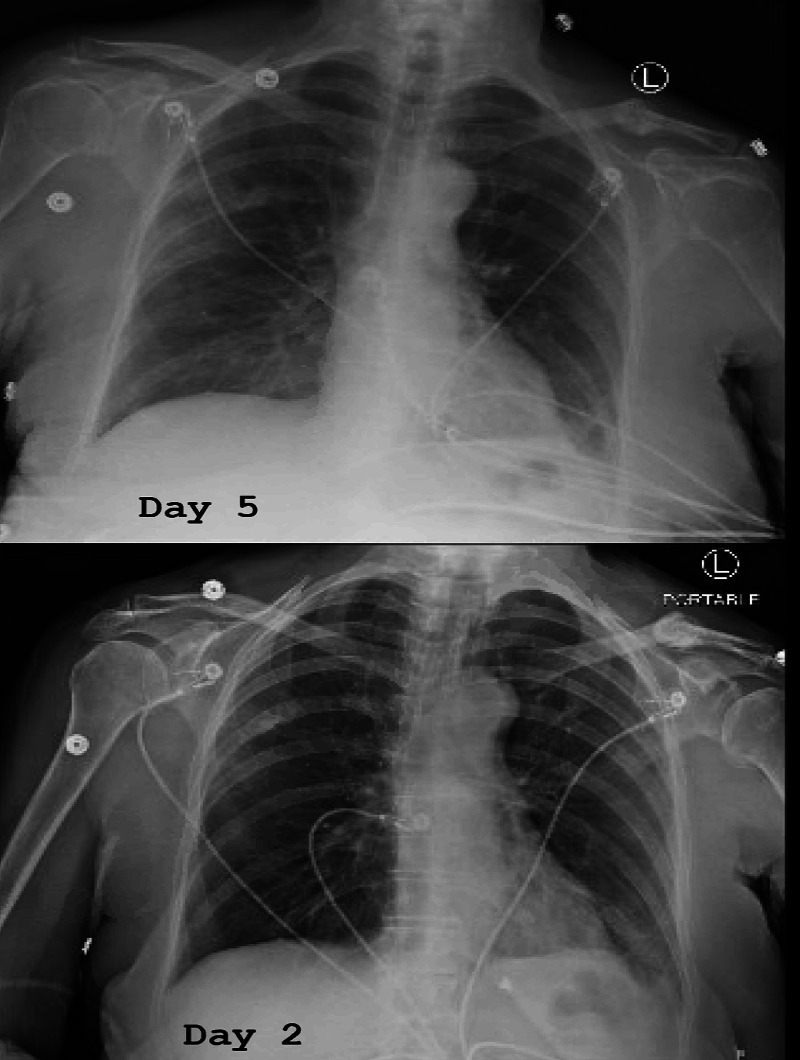
Portable CXR of the patient on day 2 and day 5 of admission CXR - chest X-ray

Our patient was managed with intravenous fluid hydration with ringers lactate alternating with normal saline, resumption of home medications, and inpatient physical therapy; however, no clinical improvement was observed with the current treatment plan. She subsequently developed febrile spikes up to a 101.3F. Repeat electrocardiogram, which was done because of increased troponin levels, was unchanged except for occasional premature ventricular contractions (PVC) but did not show any evidence of ongoing ischemia with the patient continuously denying any symptoms of chest pain. Although our patient had ill-defined hazy ground-glass opacities in the mid-upper lateral right lung - lateral left mid and lower lung respectively on day 5 chest X-ray, which was worse compared to day 2 chest X-ray, which showed poorly defined streaks in the right upper-lobe and retrocardiac left lower lobe (Figure 3) - she continued to maintain oxygen saturation above 95% on room air, did not require any oxygen supplementation and denied any symptoms of cough or shortness of breath. The patient improved remarkably with a five-day course of intravenous remdesivir therapy and a 10-day course of dexamethasone 6mg daily. Both were started on hospital day 5 of admission with notable improvement in platelet count and leucocytes in addition to normalization of troponin I and CPK levels. 

## Discussion

We just described a unique case of COVID-19 induced pancytopenia with rhabdomyolysis and viral myocarditis. Although our patient's prolonged immobilization may have aggravated her rhabdomyolysis, COVID-19 induced rhabdomyolysis has previously been reported in multiple case reports and series [8, 9]. The mechanism of SARS-CoV-2 induced rhabdomyolysis is poorly understood. Proposed mechanisms include immunologic cross-reactivity between antibodies against viral antigen and components of myocytes [10], direct invasion of myocytes via a mechanism similar to the flu virus [11], and direct effects of the amplified immune response as part of the cytokine storm-induced damage [12]. What is interesting about our case is that our patient also had evidence of viral-induced myelosuppression with pancytopenia and myocarditis. Pancytopenia in COVID-19 infection is rarely reported in the literature. Our case was self-limiting, improved significantly with the treatment of COVID-19 infection, and did not require bone marrow biopsy similar to that by Hersby et al. [13]. Our patient may have had bone marrow viral infiltration as reported in the first case of COVID-19 induced pancytopenia [14]; however, we did not pursue a biopsy given the remarkable improvement of CBC with treatment. Moreover, the most notable improvement was in the platelet count with a number of 117000/µL prior to discharge.

Immune thrombocytopenia due to COVID-19 is well described in the literature [15, 16]. While several postulated mechanisms exist, including direct infiltration of cells and platelets with defective marrow microenvironment, decreased thrombopoietin production from viral-mediated liver damage, platelet aggregation and consumption with the formation of microthrombi following pulmonary endothelial damage and immune system-mediated platelet destruction, what has been consistent across the literature is the response of COVID-19 induced thrombocytopenia to short courses of corticosteroids and intravenous immunoglobulin [16]. Our patient did receive steroids in the form of dexamethasone, and this most likely had contributed to the resolution of her thrombocytopenia.

Our patient manifested COVID-19 related myocarditis because of significantly elevated troponin I levels and PVCs that were detected on telemetry and repeat electrocardiograms (EKGs). It has been reported that 78.7% of patients with myocarditis exhibited some form of arrhythmias [17], while other reports of COVID-19 related myocarditis did have elevated troponin levels [18, 19]. It has been postulated that the SARS-CoV-2 virus gains access via the angiotensin-converting enzyme 2 (ACE2) receptors present on cardiomyocytes with concomitant disruption of the cell membrane and the conductance system resulting in leakage of cardiac enzymes and arrhythmias. ACE2 is also found in the ciliated columnar epithelial cells of the respiratory tract and type II pneumocytes [20].

To our knowledge, this is the first report of a combination of these rare presentations in a single scenario of COVID-19 infection. Others have reported isolated findings of pancytopenia, rhabdomyolysis, and myocarditis. However, our patient had a combination of all these occurring simultaneously that responded to targeted COVID-19 therapy. Notably, our patient also had evidence of worsening pulmonary infiltrates, suggesting a viral infection of the lungs, which is typical for SARS-CoV-2 infection.

While pursuing a cardiac magnetic resonance imaging, endomyocardial biopsy, diagnostic coronary angiogram, bone marrow, and skeletal muscle biopsy may have provided more specific pathologic information and severity of involvement; these studies were not necessary because of significant clinical improvement with targeted therapy and risk of contamination due to strict isolation procedures.

## Conclusions

Severe COVID-19 infection can exist without diffuse pulmonary infiltrates requiring oxygen support or ventilatory assistance. While these atypical manifestations are critical enough to warrant hospitalization, awareness is necessary, as well as the need to extend the scope of COVID-19 directed therapy beyond just subjective and objective shortness of breath to include unusual symptoms and atypical findings on the investigation in conjunction with a positive SARS-CoV-2 test. This approach may help resolve these unusual associations and hasten recovery. More emphasis should be placed on extrapulmonary pathophysiology and manifestations of COVID-19 disease to help understand and control this turbulent pandemic.
